# Supplementary material for: Surgical and transcatheter aortic valve replacement after orthotopic heart transplantation: a case series
Source: Commun Med (Lond). 2025 Oct 1;5:412. doi: 10.1038/s43856-025-01151-8 (PMC12488981; doi:10.1038/s43856-025-01151-8)
Supplement: Supplementary file 2 — Supplementary Information [file 43856_2025_1151_MOESM2_ESM.pdf]

Figure S1 immunosuppressive management

Patient	Initial immunosuppressive medication					Immunosuppressive medication prior to procedure					Management	Stop pre-OP day	Restart post-OP day
	CNI	CS	Antimet	mTORi	Other	CNI	CS	Antimet	mTORi	Other			
T1	CyA	P	AZA	-	-	CyA	P	-	EVE	-	No change	-	-
T2	CyA	P	-	-	Study Med.	TAC	P	-	-	-	No change	-	-
T3	CyA	P	MMF	-	-	CyA	-	MMF	-	-	No change	-	-
T4	TAC	P	MMF	-	-	TAC	P	-	EVE	-	No change	-	-
T5	CyA	P	MMF	-	-	TAC	P	MMF	-	-	No change	-	-
T6	***	***	***	***	***	CyA	P	-	EVE	-	EVE paused	-1	transfer POD 7
T7	CyA	P	AZA	-	-	CyA	P	-	EVE	-	EVE paused	-1	1
T8	TAC	P	MMF	-	-	TAC	P	-	-	-	No change	-	-
T9	***	***	***	***	***	CyA	P	MMF	-	-	MMF paused	-9	transfer POD 6d
S1	CyA	P	MMF	-	-	CyA	-	-	EVE	-	***	***	***
S2	CyA	P	AZA	-	-	CyA	P	AZA	-	-	AZA + P paused	-13	transfer POD 22
S3 (IE)	CyA	P	AZA	-	-	CyA	P	-	-	-	No change	-	-
S4 (IE)	CyA	P	AZA	-	-	CyA	P	AZA	-	-	AZA paused	-1	2
S5	CyA	P	AZA	-	-	CyA	P	-	EVE	-	EVE paused	-1	transfer POD 12
S6	CyA	P	MMF	-	-	CyA	P	MMF	-	-	No change	-	-
S7 (IE)	CyA	P	-	EVE	-	CyA	P	MMF	-	-	MMF paused	-1	transfer POD 4

Details:
Transfer: the patients were transfered to our partner clinic with paused medication, information on the exact restart of paused medication is not available. Medication was restarted at the time of the next outpatient visit.
Patient T9: the patient suffered from EBV-associated hepatitis after TAVR, therefore MMF remained paused for longer than 3 month unrelated to the procedure
Patient S7: MMF was paused until discharge of rehabilitation due to prolonged anti-infectious treatment for infective endocarditis
