# Supplementary material for: Surgical and transcatheter aortic valve replacement after orthotopic heart transplantation: a case series
Source: Commun Med (Lond). 2025 Oct 1;5:412. doi: 10.1038/s43856-025-01151-8 (PMC12488981; doi:10.1038/s43856-025-01151-8)
Supplement: Supplementary file 3 — Description of Additional Supplementary Files [file 43856_2025_1151_MOESM3_ESM.docx]

**Description of Additional Supplementary Files**

**Supplementary Data 1: Data regarding the heart transplantation**

Categorical data is displayed as number (%), continuous data is stated as median (interquartile range)

Abbreviations: D-R = donor - recipient (subtracted); NA = not available; BMI = body mass index; ECLS = extra-corporal circulatory life support; HTX = heart transplantation; DCM = dilated cardiomyopathy; CAD = coronary artery disease; HLA = human- leucocyte antigen; DSA= donor-specific antibodies; ISHLT = international society of heart and lung transplantation

**Supplementary Data 2: Peri-procedural details**

T1-9: TAVR patients; S1-7: SAVR patients.

NYHA = New York Heart Association class; LVEF = left ventricular ejection fraction; TAVR = transcatheter aortic valve replacement; SAVR = surgical aortic valve replacement; AV block = atrio-ventricular blockage; EBV = Epstein-Barr virus; EVANS syndrome = autoimmune disease, which commonly is associated with autoimmune hemolytic anemia (AIHA) and immune thrombocytopenia (ITP); IABP = intra-aortic balloon pump; COD = cause of death; VIS = vasoactive inotropic score; POD = postoperative day

**Supplementary Data 3: Echocardiographic specifics of the aortic valve prior to TAVR/SAVR**

Abbreviations:

Patients: T1-9 = TAVR patients, S1-7 = SAVR patients

Parameters: meanPG = mean pressure gradient over the aortic valve, maxPG = maximal pressure gradient over the aortic valve, LVEF = left ventricular ejection fraction, LVEDD = left ventricular end-diastolic diameter, AVA = aortic valve opening area, AV Vmax = aortic valve peak velocity, VC = vena contracta, PHT = pressure half time, sysPAP (echo) = systolic pulmonary artery pressure, estimated by echocardiography, RHC = right-heart catheterization, CVP = central venous pressure

Diagnosis: AS = aortic valve stenosis, AR = aortic valve regurgitation, I° = mild, II° = moderate, III° = severe, NYHA = New York Heart Association classification, LV = left ventricle, LFLG = low-flow low gradient, par LFLG = paradoxical low-flow low-gradient

**Supplementary Data 4: Source data of Figure 1 and 3**
