# Supplementary material for: Surgical and transcatheter aortic valve replacement after orthotopic heart transplantation: a case series
Source: Commun Med (Lond). 2025 Oct 1;5:412. doi: 10.1038/s43856-025-01151-8 (PMC12488981; doi:10.1038/s43856-025-01151-8)
Supplement: Supplementary file 4 — Supplementary Data 1 [file 43856_2025_1151_MOESM4_ESM.docx]

| **Supplementary Data 1: Data regarding the heart transplantation** | | | |
| --- | --- | --- | --- |
| **Parameters** | **All** | **TAVR** | **SAVR** |
| Donor weight (kg) | 75 (65; 80) | 72.5 (64; 78.5) | 80 (67.5; 80) |
| Recipient weight (kg) | 66 (56; 86.5) | 63 (56.5; 67.25) | 85 (59.5; 88) |
| D-R weight difference (kg)   - donor > recipient - donor < recipient - missmatch < 5kg - NA | 5.0 (-5.5; 13.5)  8 (50%)  5 (31%)  2 (13%)  1 (6%) | 13.00 (0.25; 20.75)  5 (56%)  2 (22%)  1 (11%)  1 (11%) | 4 (-8; 5)  3 (43%)  3 (43%)  1 (14%)  0 (0%) |
| Donor height (cm) | 170 (167; 178) | 169 (165; 173) | 170 (170; 180) |
| Recipient height (cm) | 170 (166; 179) | 168 (164; 173) | 175 (171; 179) |
| D-R height difference (cm)   - donor > recipient - donor < recipient - missmatch < 5cm - NA | 0.0 (-7.5; 6.5)  5 (31%)  6 (38%)  4 (25%)  1 (6%) | 0.5 (-6.5; 3.0)  2 (23%)  3 (33%)  3 (33%)  1 (11%) | -2.0 (-7.5; 6.5)  3 (43%)  3 (43%)  1 (14%)  0 (0%) |
| Donor BMI (kg/m^2^) | 25 (22.5; 27.5) | 26 (23.5; 27.25) | 25 (22.5; 27) |
| Recipient BMI (kg/m^2^) | 23 (18.5; 27) | 22.5 (18.75; 25.25) | 23 (20.5; 28) |
| D-R BMI difference (kg/m^2^)   - donor > recipient - donor < recipient - missmatch < 5 kg/m^2^ - NA | 2 (0; 4)  3 (19%)  2 (12%)  10 (63%)  1 (6%) | 3.50 (-0.25; 9.00)  3 (33%)  1 (11%)  4 (45%)  1 (11%) | 0 (0; 2.5)  0 (0%)  1 (14%)  6 (86%)  0 (0%) |
| Gender-matching D/R   - male-male - male-female - female-male - female-female - NA | 5 (31%)  1 (6%)  5 (31%)  4 (25%)  1 (6%) | 2 (22%)  1 (11%)  2 (22%)  3 (33%)  1 (11%) | 3 (43%)  0 (0%)  3 (43%)  1 (14%)  0 (0%) |
| Donor age (years) | 52 (49.5; 58) | 54 (52; 61.75) | 49 (46; 51) |
| Recipient age (years) | 56.5 (44; 59.5) | 56 (44; 59) | 57 (46; 65.5) |
| D-R age difference   - donor > recipient - donor < recipient - missmatch < 5 years - NA | 0 (-7; 12)  5 (31%)  5 (31%)  5 (31%)  1 (6%) | 5.5 (-1.75; 13)  4 (45%)  1 (11%)  3 (33%)  1 (11%) | -7 (-7.5; 1.5)  1 (14%)  4 (57%)  2 (29%)  0 (0%) |
| Anastomosis of HTX   - Biatrial - Bicaval | 13 (81%)  3 (19%) | 6 (67%)  3 (33%) | 7 (100%)  0 (0%) |
| Ischemic time (min) | 203 (179; 240) | 202 (187; 220) | 207 (171.5; 240) |
| Reperfusion time | 127 (90; 212) | 144 (82; 210) | 110 (92.5; 190) |
| Cardiac surgery prior to HTX   - Yes - No | 8 (50%)  8 (50%) | 5 (56%)  4 (44%) | 3 (43%)  4 (57%) |
| Cardiac disease   - DCM - CAD - Other | 12 (74%)  2 (13%)  2 (13%) | 8 (89%)  0 (0%)  1 (11%) | 4 (57%)  2 (29%)  1 (14%) |
| HLA antibodies  Class I   - negative - DSA - Non-DSA   Class II   - negative - II DSA - II Non-DSA   No donor specification avaible  NA | 5 (31%)  2 (12%)  3 (19%)  5 (31%)  2 (12%)  3 (19%)  1 (6%)  5 (31%) | 4 (44%)  1 (11%)  3 (33%)  4 (44%)  1 (11%)  3 (33%)  1 (11%)  0 (0%) | 1 (14%)  1 (14%)  0 (0%)  1 (14%)  1 (14%)  0 (0%)  0 (0%)  5 (72%) |
| Biopsy proven rejection in past  medical history   - None - 1R (ISHLT) - ≥2R (ISHLT) | 6 (38%)  8 (50%)  4 (25%) | 4 (44%)  4 (44%)  3 (33%) | 2 (29%)  4 (57%)  1 (14%) |
| Cardiac allograft vasculopathy  No  Yes   - without intervention - 1 intervention - ≥ 2 interventions | 7 (44%)  9 (56%)  5 (31%)  2 (13%)  2 (13%) | 4 (44%)  4 (44%)  1 (11%)  1 (11%)  2 (22%) | 3 (43%)  5 (71%)  4 (57%)  1 (14%)  0 (0%) |

Categorical data is displayed as number (%), continuous data is stated as median (interquartile range)

Abbreviations: D-R = donor - recipient (subtracted); NA = not available; BMI = body mass index; ECLS = extra-corporal circulatory life support; HTX = heart transplantation; DCM = dilated cardiomyopathy; CAD = coronary artery disease; HLA = human- leucocyte antigen; DSA= donor-specific antibodies; ISHLT = international society of heart and lung transplantation;
