# Supplementary material for: Surgical and transcatheter aortic valve replacement after orthotopic heart transplantation: a case series
Source: Commun Med (Lond). 2025 Oct 1;5:412. doi: 10.1038/s43856-025-01151-8 (PMC12488981; doi:10.1038/s43856-025-01151-8)
Supplement: Supplementary file 5 — Supplementary Data 2 [file 43856_2025_1151_MOESM5_ESM.docx]

| **Supplementary Data 2: Peri-procedural details** | | | | | |
| --- | --- | --- | --- | --- | --- |
| **PID** | **Years after HTX** | **Preoperative condition** | **Postoperative course** | **Outcome** | **Survival** |
| **T1** | 25 | - NYHA III - LVEF 20 % - peripheral edema - ascites - new-onset dialysis due to acute on chronic renal failure - VIS 0   Sapien-3  (Edwards Lifesciences)  26mm | - prolonged need of inotropic medication - catheter-associated infection - cardiopulmonary resuscitation due to ventricular fibrillation during implantation of a dialysis catheter | The patient died postoperatively due to septic multi-organ failure | 83 days (deceased)  COD: sepsis caused by spontaneous bacterial peritonitis |
| **T2** | 16 | - NYHA III - LVEF 60 % - recurrent pleural effusions - post-capillary pulmonary hypertension caused by a paradoxical low-flow, low-gradient aortic valve stenosis - VIS 0   Evolut R  (Medtronic)  29mm | - stroke with impaired vigilance, structural seizures and need of prolonged mechanical ventilation - ventilator-associated sepsis | The patient died during neurological rehabilitation. | 62 days (deceased)  COD: unknown |
| **T3** | 17 | - NYHA IV - LVEF 55 % - progressive peripheral edema - AV Blockage II with need of pacemaker implantation - progressive respiratory failure with need of mechanical ventilation - VIS 0   Sapien-3  (Edwards Lifesciences)  26mm | - pneumogenic sepsis - new-onset dialysis due to acute on chronic renal failure | The patient died postoperatively due to septic multi-organ failure | 41 days (deceased)  COD: sepsis |
| **T4** | 16 | - NYHA III - LVEF 55 % - No signs of acute decompensation - VIS 0   Sapien^3^ ultra  (Edwards Lifesciences)  23mm | - uneventful | Discharged on POD 5  with subjective improvement | 535 days (alive) |
| **T5** | 10 | - NYHA II - LVEF 41 % - light edema - VIS 0   Sapien-3  (Edwards Lifesciences)  29mm | - uneventful | Discharged on POD 10  with subjective improvement | 2145 days (alive) |
| **T6** | 5 | - NYHA III - LVEF 60 % - worsening of functional performance - no signs of acute decompensation - VIS 0   Evolut R  (Medtronic)  26mm | - pacemaker implantation due to AV blockage III° (POD 5) | Discharged on POD 31  with subjective improvement | 402 days (deceased)  COD: metastasized ovarian cancer |
| **T7** | 20 | - NYHA III - LVEF 55 % - worsening of functional performance - no signs of acute decompensation - VIS 0   Sapien^3^ ultra  (Edwards Lifesciences)  23mm | uneventful | Discharged on POD 3  with subjective improvement | 889 days (alive) |
| **T8** | 2 | - NYHA III - LVEF 60 % - worsening of functional performance - no signs of acute decompensation - progressive dilation of the left ventricle - VIS 0   JenaValve  25mm | uneventful | Discharged on POD 19  with subjective improvement  The patient underwent successful Re-TAVR valve-in-valve due to degeneration of aortic valve prothesis 9 years after the first TAVR | 3879 days (alive) |
| **T9** | 17 | - NYHA III - LVEF 30 % - no signs of acute decompensation - VIS 0   Sapien-3  (Edwards Lifescience)  29mm | - No procedure-related complications, but complicative and prolonged hospital stay | Discharged on POD 96  Early prosthetic valve endocarditis with conservative, permanent antibiotic treatment | 426 days (deceased)  COD: endocarditis |
| **S1** | 17 | - NYHA IV - LVEF 55 % - acute decompensation due to multiple valve dysfunction: triple valve replacement - VIS unknown   Carpentier-Edwards PERIMOUNT Magna Ease aortic valve  (Edwards Lifesciences)  23mm | - delayed sternum closure due to bleeding disorder - postoperative extra-corporal circulatory support for 5 days - new-onset dialysis - sepsis with positive blood cultures | Discharge on  POD 97 | 828 days (deceased)  COD: acute graft failure |
| **S2** | 9 | - NYHA IV - LVEF 30 % - severe decompensation with cardiogenic shock and - pneumonia with respiratory failure and need of mechanical ventilation - VIS 42   St Jude Medical 23mm | - uneventful | Discharged on POD 97  with subjective improvement | 444 days (deceased)  COD: complicated upper GI-bleed caused by an ischemic duodenal ulcer caused by severe arteriosclerotic disease. |
| **S3** | 4 | - NYHA III - LVEF 60 % - recurrence of a conservatively treated infective endocarditis with a methicillin-resistant staphylococcus - slight peripheral edema - VIS 14   CARBOMEDICS^TM^  (CORCYM)  27mm | uneventful | Discharged on POD 19  with subjective improvement. | 534 days (deceased)  COD: metastasized adenocarcinoma of the stomach |
| **S4** | 1 | - NYHA II - LVEF 65 % - Asymptomatic - progressive dilation of the ventricle - culture-negative infective endocarditis - VIS 0   Hancock T 505  (Medtronic)  25mm | uneventful | Discharged on POD 12  - re-aortic valve replacement due to late prosthetic endocarditis 10 years after SAVR.  - re-re-aortic valve replacement with concomitant coronary artery bypass surgery after 7 years due to re-endocarditis | 6022 days (deceased)  COD: Sepsis after third aortic valve replacement |
| **S5** | 8 | - NYHA IV - LVEF 60 % - cardiac decompensation with pulmonary edema and acute on chronic renal failure - VIS 0   St Jude Medical 21mm | - new-onset permanent dialysis due to acute-on-chronic renal failure - prolonged stay due to need of recompensation | Discharged on POD 23  Re-admission and re-thoracotomy due to relevant hematoma behind the right atrium | 1112 days (deceased)  COD: unknown |
| **S6** | 1 | - NYHA II - LVEF 60 % - No further specifics regarding the preoperative condition available - VIS 0   ADVANTAGE mechanical heart valve  (Medtronic)  23mm | - postoperative hemodynamic deterioration with secondary implantation of an IABP, explantation after 4 days - prolonged need of mechanical ventilation - postoperative stroke with left-sided hemiparesis | Discharged on POD 37  with regressive neurological deficits. | 518 days (deceased)  COD: acute graft failure complicated by sepsis |
| **S7** | 18 | - NYHA II - LVEF 60 % - infective endocarditis with Enterococcus faecalis - VIS 0   Carpentier-Edwards PERIMOUNT Magna Ease aortic valve  (Edwards Lifesciences)  23mm | - uneventful | Discharged on POD 27 | 4 days (alive) |

T1-9: TAVR patients; S1-7: SAVR patients.

NYHA = New York Heart Association class; LVEF = left ventricular ejection fraction; TAVR = transcatheter aortic valve replacement; SAVR = surgical aortic valve replacement; AV block = atrio-ventricular blockage; EBV = Epstein-Barr virus; EVANS syndrome = autoimmune disease, which commonly is associated with autoimmune hemolytic anemia (AIHA) and immune thrombocytopenia (ITP); IABP = intra-aortic balloon pump; COD = cause of death; VIS = vasoactive inotropic score; POD = postoperative day
