# Supplementary material for: Surgical and transcatheter aortic valve replacement after orthotopic heart transplantation: a case series
Source: Commun Med (Lond). 2025 Oct 1;5:412. doi: 10.1038/s43856-025-01151-8 (PMC12488981; doi:10.1038/s43856-025-01151-8)
Supplement: Supplementary file 6 — Supplementary Data 3 [file 43856_2025_1151_MOESM6_ESM.docx]

| **Supplementary Data 3:** **Echocardiographic specifics of the aortic valve prior to TAVR/SAVR** | | | | | | | | | | | | |
| --- | --- | --- | --- | --- | --- | --- | --- | --- | --- | --- | --- | --- |
| **Case** | **Diagnosis** | **Indication for procedure** | **meanPG** | **maxPG** | **LVEF** | **LVEDD** | **AVA** | **AV Vmax** | **VC** | **PHT** | **sysPAP (echo)** | **PAP (RHC)** |
|  |  |  | **(mmHg)** | **(mmHg)** | **(%)** | **(mm)** | **(cm^2^)** | **(m/s)** | **(mm)** | **(ms)** | **(mmHg)** | **(mmHg)** |
| T1 | AS III°  LFLG | Symptomatic patient NYHA III  Progressive worsening of LVEF Progressive dilation of LV | 15 | 24 | 20 | 61 | 1.3 | 2.4 |  |  | 40  + CVP |  |
| T2 | AS III°  par. LFLG | Symptomatic patient NYHA III  Repeated hydropic decompensation | 12 | 20 | 60 | 39 | 1.4 | 2.2 |  | 480 | 49  + CVP |  |
| T3 | AI III°  AS II° | Symptomatic patient NYHA IV  Progressive decompensation  Progressive dilation of LV  AVA < 1 cm^2^ | 22 | 45 | 55 | 41 | 0.92 | 3.4 | 3.9 |  | 50  + CVP |  |
| T4 | AI II°  AS III° | Symptomatic patient NYHA III  AVA < 1 cm^2^ | 40 |  | 55 | 53 | 0.76 | 4.0 |  | 450 | 24  + CVP |  |
| T5 | AS III° | Symptomatic patient NYHA II  Positive stress echo  AVA < 1 cm^2^ | 19 | 31 | 41 | 52 | 0.89 |  |  |  | 29  + CVP |  |
| T6 | AI I°  AS III° | Symptomatic patient NYHA III  meanPG > 40mmHg  AVA < 1 cm^2^ | 48 | 78 | 60 | 39 | 0.72 | 4.4 |  |  | 37  + CVP |  |
| T7 | AI II°  AS III°  par. LFLG | Symptomatic patient NYHA III  AVA < 1 cm^2^ | 39 |  | 55 | 31 | 0.72 |  |  | 332 | 41  + CVP |  |
| T8 | AI III° | Symptomatic patient NYHA III  Progressive dilation of LV |  |  | 60 | 53 |  |  |  |  | 45  + CVP |  |
| T9 | AS III°  LFLG | Symptomatic patient NYHA III  Progressive dilation of LV | 30 | 43 | 30 | 53 |  |  |  |  |  | 65/10/20 |

| S1 | AI III° | Symptomatic patient NYHA IV  Repeated hydropic decompensation  Regurgitation of aortic, mitral and tricuspid valves |  |  | 55 | 46 | 2 |  | 5 | 221 |  | 27/11/18 |
| --- | --- | --- | --- | --- | --- | --- | --- | --- | --- | --- | --- | --- |
| S2 | AS III°  LFLG | Symptomatic patient NYHA IV  Severe hydropic decompensation | 32 | 49 | 30 | 47 | 1 |  |  |  |  | 45/25/36 |
| S3 | AI III° (IE) | Symptomatic patient NYHA III  Infective endocarditis |  |  | 60 |  |  |  |  |  |  | 28/12/17 |
| S4 | AI III° (IE) | Symptomatic patient NYHA II  Infective endocarditis  Progressive dilation of LV |  |  | 65 | 66 |  | 5.6 |  |  | 45  + CVP |  |
| S5 | AS III° | Symptomatic patient NYHA IV  meanPG > 40mmHg | 48 | 81 | 60 | 50 |  |  |  |  |  | 33/16/22 |
| S6 | AI I°,  AS III° | Symptomatic patient NYHA II  meanPG > 40mmHg  Progressive dilation of LV | 52 | 80 | 60 | 59 |  |  |  |  | 70  + CVP |  |
| S7 | AI II° (IE) | Symptomatic patient NYHA II  Infective endocarditis | 17 | 28 | 60 | 56 |  | 3.15 |  | 272 | 27  + CVP |  |

Abbreviations:

Patients: T1-9 = TAVR patients, S1-7 = SAVR patients

Parameters: meanPG = mean pressure gradient over the aortic valve, maxPG = maximal pressure gradient over the aortic valve, LVEF = left ventricular ejection fraction, LVEDD = left ventricular end-diastolic diameter, AVA = aortic valve opening area, AV Vmax = aortic valve peak velocity, VC = vena contracta, PHT = pressure half time, sysPAP (echo) = systolic pulmonary artery pressure, estimated by echocardiography, RHC = right-heart catheterization, CVP = central venous pressure

Diagnosis: AS = aortic valve stenosis, AR = aortic valve regurgitation, I° = mild, II° = moderate, III° = severe, NYHA = New York Heart Association classification, LV = left ventricle, LFLG = low-flow low gradient, par LFLG = paradoxical low-flow low-gradient
